# Indomethacin reduces rates of aortic dissection and rupture of the abdominal aorta by inhibiting monocyte/macrophage accumulation in a murine model

**DOI:** 10.1038/s41598-019-46673-z

**Published:** 2019-07-24

**Authors:** Shota Tomida, Kenichi Aizawa, Norifumi Nishida, Hiroki Aoki, Yasushi Imai, Ryozo Nagai, Toru Suzuki

**Affiliations:** 1 Department of Clinical Pharmacology, Jichi Medical University, Tochigi, Japan; 2 Division of Cardiovascular Medicine, Department of Internal Medicine, Kurume University School of Medicine, Kurume, Fukuoka Japan; 3 Cardiovascular Research Institute, Kurume University, Kurume, Japan; 4 Department of Cardiovascular Sciences, University of Leicester, Cardiovascular Research Centre, Leicester, UK; 5 National Institute for Health Research Leicester Cardiovascular Biomedical Research Unit, Glenfield Hospital, Leicester, UK; 6 Jichi Medical University, Tochigi, Japan

**Keywords:** Aortic diseases, Drug discovery, Cardiology, Experimental models of disease

## Abstract

Aortic dissection is a life-threatening condition, which is characterised by separation of the constituent layers of the aortic wall. We have recently shown that monocyte/macrophage infiltration into the aortic wall is a pathogenic mechanism of the condition. In the present study, we investigated whether the anti-inflammatory agent, indomethacin, could inhibit monocyte/macrophage accumulation in the aortic wall and ensuing dissection. Indomethacin was administered (from 3 days prior with daily oral administration) to mice in which aortic dissection was induced using beta-aminopropionitrile (BAPN) and angiotensin II (Ang II) infusion (2 weeks). Indomethacin prevented death from abdominal aortic dissection and decreased incidence of aortic dissection by as high as 40%. Histological and flow cytometry analyses showed that indomethacin administration resulted in inhibition of monocyte transendothelial migration and monocyte/macrophage accumulation in the aortic wall. These results indicate that indomethacin administration reduces rate of onset of aortic dissection in a murine model of the condition.

## Introduction

Aortic dissection is characterized by separation of the layers that compose the aortic wall^1,2^. A tear in the intima of the aorta results in blood flow to reach the medial layer, causing separation of the layers of the aortic wall^2^. This separation of the layers continues to weaken the aortic wall which can lead to aortic rupture or malperfusion of end-organs, either which can have fatal outcomes if untreated^3^.

In the acute setting, dissections of the ascending aorta are generally treated surgically, and those of the descending aorta are treated medically (anti-impulse therapy) with additional stent therapy in complicated cases.

A lysyl oxidase inhibitor, beta aminopropionitrile inhibits the collagen fiber cross-linkage, and in combination with angiotensin II, has been shown to induce aortic dissection in mice and rats. Various routes of administration including oral gavage^4^, subcutaneous osmotic pump infusion^5,6^, and subcutaneous injection^7^ have previously been established.

A growing number of recent studies have shown the involvement of inflammation in the pathogenesis of aortic dissection^1,4,8^. We previously demonstrated that infiltration of macrophages in the aorta coupled with local and/or systemic granulocyte-macrophage colony-stimulating factor (GM-CSF) upregulation is essential for onset of aortic dissection in a murine model involving direct application of calcium chloride to the aorta and continuous administration of angiotensin II (Ang II)^1^. Others have also shown involvement of granulocytes and granulocyte stimulation factor (G-CSF) in onset of aortic dissection and subsequent aortic rupture^4^. Further, T helper 17 cells (Th17 cells), characterized by their ability to produce interleukin 17 and mediate inflammation, have also been shown to promote development of aortic dissection^8^. Collectively, inflammatory mechanisms contribute to the onset of aortic dissection in animal disease models.

The involvement of inflammatory cytokines and cells to onset of aortic dissection suggests the possibility of anti-inflammatory therapies as prevention/treatment against aortic dissection. In the present study, we investigated whether the anti-inflammatory agent, indomethacin, prevents aortic dissection in a murine model of the condition.

## Results

### Indomethacin administration decreases rates of abdominal aortic dissection and rupture

Initial pilot studies in 13 mice were done to determine appropriate dosage (6, 12 and 24 mg/L (n = 5, 4, and 4, respectively), and the group given 12 mg/L showed the highest survival rate. Mortality without aortic rupture was not observed. Organs from all mice including those that developed aortic dissection did not show changes in visual morphology. Loss of weight, appetite, and activity was not observed in the mice free from aortic dissection (data not shown).

**Figure 1:**
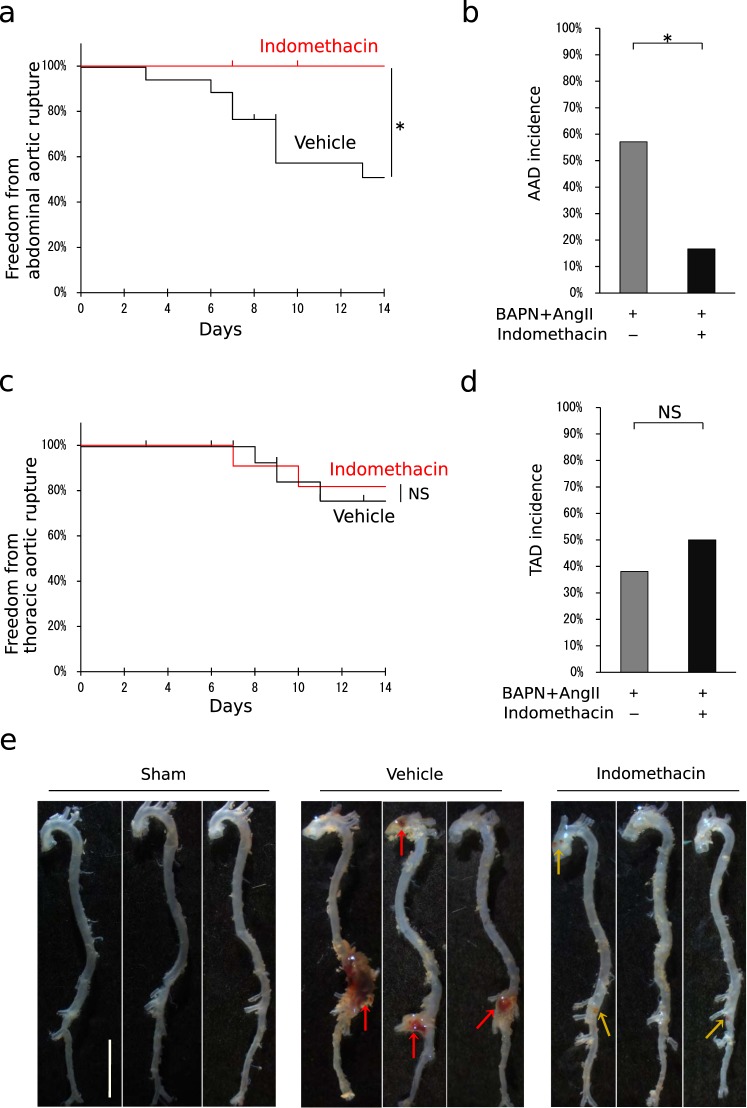
Indomethacin protects from aortic dissection and rupture. (**a,c**) Survival curves of the mice infused with beta-aminopropionitrile (BAPN) and angiotensin II (Ang II) in addition to administration of either indomethacin (n = 12) or vehicle (n = 16) when only death involving (**a**) abdominal aortic rupture or (**c**) thoracic aortic rupture was counted as death. The vertical ticks on the curves indicate withdrawal of mice whose causes of death were not (**a**) abdominal aortic rupture or (**c**) thoracic aortic rupture. Log-rank test. *P < 0.05 (**b,d**) Incidence rate of (**b**) abdominal aortic dissection (AAD) or (**d**) thoracic aortic dissection (TAD). Dilatation and/or haematomas in the aorta after 2 weeks of infusion or aortic rupture were observed. Vehicle group, n = 15. Indomethacin group, n = 12. Fisher’s exact test. *P < 0.05 (**e**) Representative photographs of aortas after 2 weeks of infusion of water and 10 mM acetic acid (sham), BAPN/Ang II infusion and vehicle administration, or BAPN/Ang II infusion and indomethacin administration. Note that in the vehicle group, the aorta showed increased diameters and major haematomas (red arrows) although the aorta from the indomethacin group, on the other hand, exhibited smaller haematomas (yellow arrows). Scale bar: 5 mm.

In the full-scale study, mice were administered beta-aminopropionitrile (BAPN) and angiotensin II (Ang II) to induce aortic dissection, and simultaneously administered with indomethacin or vehicle for 2 weeks. Fatal abdominal aortic rupture, one of the complications of aortic dissection, was compared between the indomethacin-administered group and the vehicle-administered group. Administration of indomethacin was associated with significant improvement in the survival rate. Mice infused with BAPN/Ang II without indomethacin administration died of aortic rupture as early as on the third day and the survival rate dropped to approximately 40% in 2 weeks, whereas with indomethacin administration, none of the mice developed abdominal aortic rupture (Fig. 1a). Similarly, the incidence of aortic dissection was 85% in mice treated with vehicle, whereas mice treated with indomethacin showed approximately 0.6 times as high incidence rate as those treated with vehicle (Fig. 1b). On the other hand, indomethacin administration did not significantly improve survival from thoracic aortic rupture. Without indomethacin administration, the survival rate of mice infused with BAPN and Ang II was approximately 80% in 2 weeks (Fig. 1c). Significant decrease in the incidence of thoracic aortic dissection was not observed. Approximately 40% of BAPN/Ang II infused mice treated with vehicle and 50% of BAPN/Ang II infused mice treated with indomethacin showed pathological signs of aortic dissection (Fig. 1d). Furthermore, aortas from the indomethacin administered mice exhibited smaller haematomas, whereas aortas from the vehicle administered mice showed increased diameters and major haematomas (Fig. 1e). Since the preventive effect of indomethacin was less pronounced in thoracic aortas, we have hereafter focused our attention to that in abdominal aortas.

### Indomethacin administration suppresses monocyte/macrophage accumulation in the aortic wall

**Figure 2:**
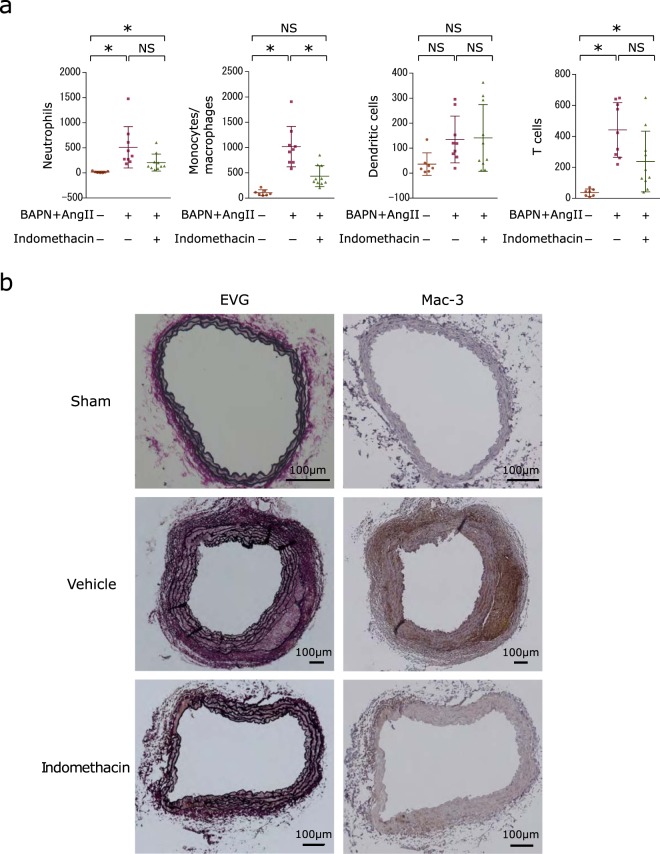
Indomethacin reduces monocyte/macrophage population in the abdominal aortic wall. (**a**) Scatter dot plots showing the means ± SD of the absolute number of neutrophils, monocytes/macrophages, DCs, or T cells measured by flow cytometry after 7 days of BAPN and Ang II infusion with or without indomethacin administration. Sham group: n = 7. Vehicle group: n = 9. Indomethacin group: n = 10. Kruskal-Wallis test followed by Dunn’s multiple comparison test. *P < 0.05 (**b**) Representative images of Elastica Van Gieson staining and immunostaining of Mac-3 in the abdominal aorta from sham, vehicle-administered, and indomethacin-administered groups after 14 days of BAPN and Ang II infusion with or without indomethacin administration (n = 5). Brown staining indicates Mac-3 positive monocytes/macrophages. Sections were counterstained with haematoxylin.

Macrophages^1^, neutrophils^4^ and Th17 cells^8^ have reported roles in development and progression of aortic dissection. In order to understand the mechanism in which indomethacin protects against aortic dissection, we compared the proportion of each cell type in the aortic wall using flow cytometry after 7 days of infusion. Mice were euthanised at 7 days since considerably more mice in the vehicle-administered group had been fatal compared to the indomethacin-administered group at this time-point as shown in Fig. 1a. Neutrophil and T cell accumulation was observed in both vehicle-treated and indomethacin-treated mice. The abdominal aortic wall from mice infused with BAPN/Ang II showed an increase in the number of monocytes/macrophages (lymphocyte antigen 6 complex locus G6D (Ly6G)^−^ CD11b^+^ CD45^+^). With indomethacin administration, BAPN/Ang II-infused mice exhibited fewer monocytes/macrophages, whereas the number of neutrophils, dendritic cells, or T cells did not significantly increase or decrease (Fig. 2a and Supplementary Fig. 1). This indicates that indomethacin administration is associated with mitigation of abdominal aortic dissection progression and reduction of monocyte/macrophage accumulation after the progression of the aortic dissection cascade marked by neutrophil accumulation^4^. Histologically, monocytes/macrophages were observed abundantly in the aortic wall of the BAPN/Ang II-infused mice that did not receive indomethacin administration. On the other hand, with indomethacin administration, monocyte/macrophage accumulation was either not observed or limited within the aortic wall (Fig. 2b).

### Abrogation of monocytes/macrophages reduces rates of aortic dissection and rupture

**Figure 3:**
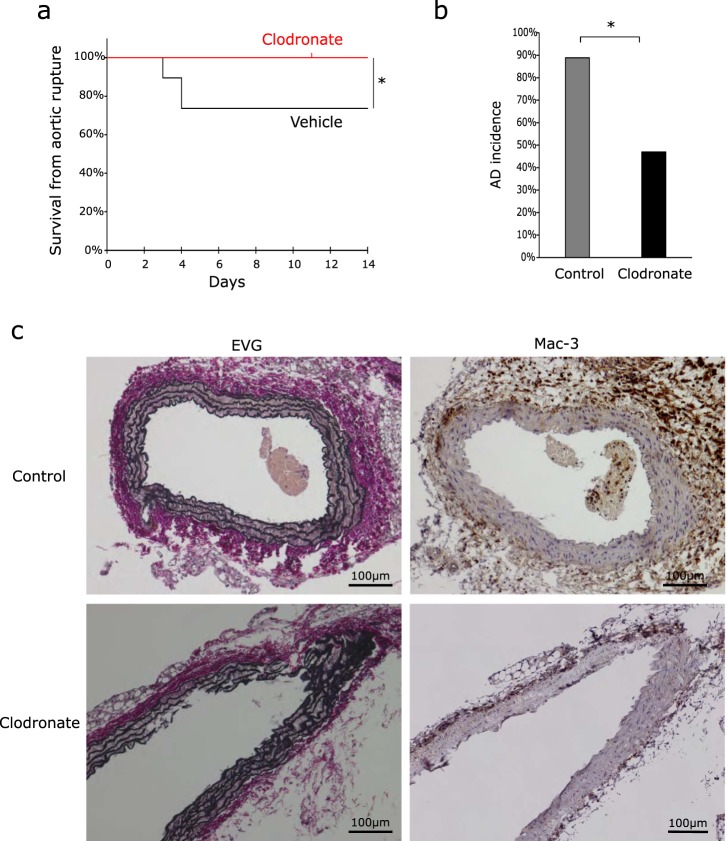
Monocyte/macrophage abrogation by clodronate attenuates aortic dissection. (**a**) Survival curves of mice that received BAPN/Ang II infusion and clodronate liposome (n = 15) or control liposome (n = 19). The vertical ticks on the curves indicate withdrawal of mice whose causes of death were not aortic rupture. Log-rank test. *P < 0.05 (**b**) Incidence rate of aortic dissection (AD). Dilatation and/or haematomas in the aorta after 2 weeks of infusion or aortic rupture were observed. Control-liposome group, n = 19. Clodronate-liposome group, n = 15. Fisher’s exact test. *P < 0.05 (**c**) Representative images of Elastica Van Gieson staining and immunostaining of Mac-3 in the abdominal aorta from control liposome and clodronate liposome groups after 14 days of BAPN and Ang II infusion (n = 3). Brown staining indicates Mac-3 positive monocytes/macrophages. Sections were counterstained with haematoxylin.

In the models of aortic dissection that were previously investigated^1,9^, macrophage abrogation by clodronate liposome injection was shown to prevent aortic dissection. In order to test whether reduction in the monocyte/macrophage population results in prevention of aortic dissection in the present model, we treated mice with clodronate liposome 2 days before and 7 days after the initiation of BAPN/Ang II infusion. Approximately 30% of the mice treated with control liposome died of aortic rupture after 2 weeks of BAPN/Ang II infusion; on the other hand, none of the mice treated with clodronate liposome developed aortic rupture (Fig. 3a). Moreover, the incidence rate of aortic dissection in BAPN/Ang II infused mice treated with clodronate liposome was 50% of that in mice treated with control liposome after 2 weeks of BAPN/Ang II treatment (Fig. 3b). Histologically, more macrophage-3 antigen (Mac-3) positive cells (monocytes/macrophages) accumulated inside the aortic wall and outside the adventitia of the control-liposome treated group. On the other hand, the clodronate-liposome treated group showed localised accumulation inside the aortic wall (Fig. 3c). These results suggest that reduction of the monocyte/macrophage population in the aorta is associated with reduced rates of aortic dissection and rupture in the present model.

### Indomethacin administration promotes accumulation of monocytes in peripheral blood

**Figure 4:**
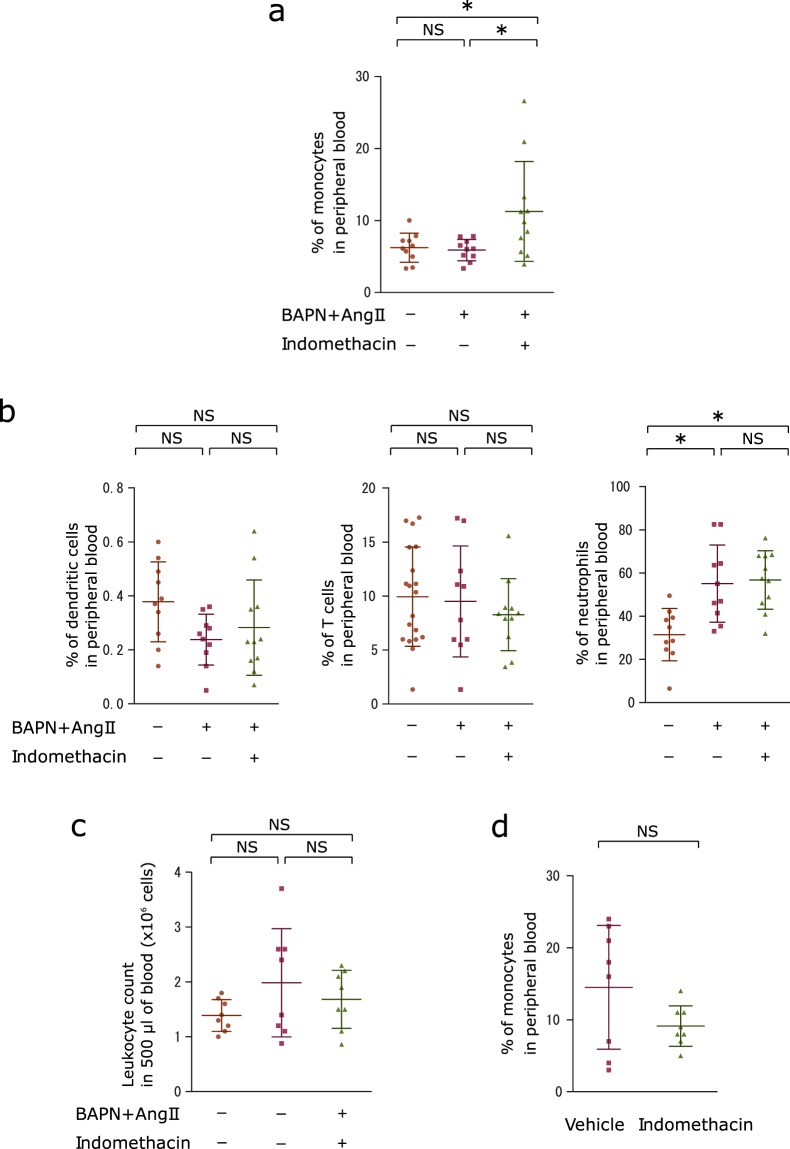
Indomethacin administration induces accumulation of monocytes in peripheral blood. (**a**) Scatter dot plots showing the means ± SD of percentages of peripheral blood CD45^+^ CD11b^+^ CD11c^−^ Ly6G^−^ monocytes in the sham (n = 10), vehicle-administered (n = 9), and indomethacin-administered (n = 11) groups after 7 days of BAPN and Ang II infusion with or without indomethacin administration. One-way ANOVA with Tukey’s multiple comparisons test. *P < 0.05 (**b**) Scatter dot plots showing the means ± SD of percentages of neutrophils (CD45^+^ CD11b^+^ Ly6G^+^), dendritic cells (CD45^+^ CD11b^−^ CD11c^+^), or T cells (CD45^+^ CD3^+^) in peripheral blood from sham (n = 10), vehicle-administered (n = 9), and indomethacin-administered (n = 11) groups after 7 days of BAPN and Ang II infusion with or without indomethacin administration. One-way ANOVA with Tukey’s multiple comparisons test. *P < 0.05 (**c**) Scatter dot plots showing the numbers of leukocytes in 500 μl of peripheral blood (n = 8). One-way ANOVA with Tukey’s multiple comparison test. (**d**) Scatter dot plots showing the means ± SD of percentage of monocytes in peripheral blood from the mice that received vehicle or indomethacin administration for 7 days (n = 8). Welch’s T test. *P < 0.05.

Since peripheral blood monocytes have been reported to be recruited to the aortic wall in response to Ang II stimulation in model mice of aortic aneurysm^10^, we tested whether the recruitment of peripheral blood monocytes to the aortic wall is blocked by indomethacin administration in the present model. Flow cytometry analysis showed that indomethacin administration doubled the population of peripheral blood monocytes in mice infused with BAPN/Ang II compared to those that did not receive indomethacin administration (Fig. 4a and Supplementary Fig. 2). Even though the neutrophil population in peripheral blood increased after BAPN/Ang II stimulation, indomethacin administration showed no effect on the neutrophil population (Fig. 4b and Supplementary Fig. 2). BAPN/Ang II infusion with indomethacin administration did not show effect on peripheral blood dendritic cells or T cell populations (Fig. 4b and Supplementary Fig. 2). This suggests that increase in the monocyte population is not due to decrease in neutrophils, dendritic cells or T cells. To test whether indomethacin by itself was capable of increasing the monocyte population, we administered indomethacin to mice for a week without BAPN or Ang II infusion. Vehicle administration and indomethacin administration in BAPN/Ang II infused mice did not alter the number of leukocytes from 500 µl of peripheral blood compared to those from sham mice (Fig. 4c). The proportion of the monocyte population did not increase after a week of indomethacin administration without BAPN or Ang II infusion (Fig. 4d). Hence, the increase in the peripheral blood monocyte population observed after a week of BAPN/Ang II infusion with indomethacin administration was not caused by indomethacin’s capability to induce proliferation or recruitment of monocytes to peripheral blood.

### Indomethacin attenuates transendothelial migration of monocytes

**Figure 5:**
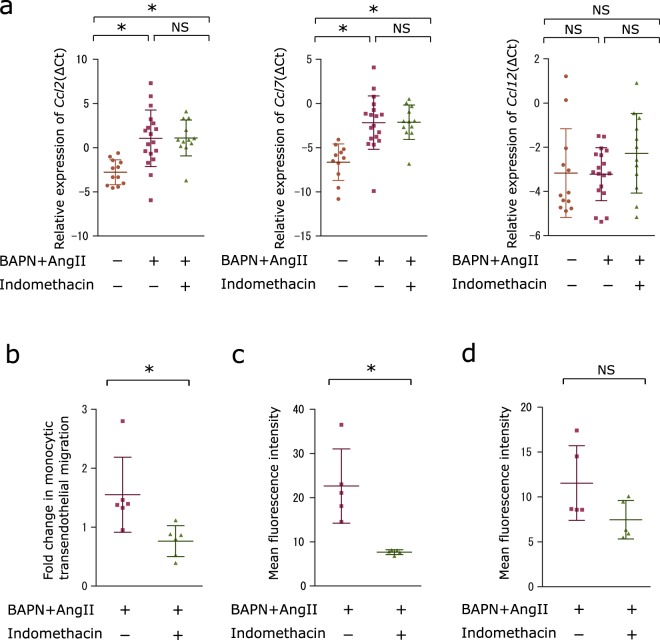
Indomethacin attenuates transendothelial migration of monocytes. (**a**) Scatter dot plots showing the means ± SD of *Ccl2, Ccl7, and Ccl12* mRNA expressions in ΔCt in the aortas from sham (n = 12), BAPN and Ang II infused (n = 18), or BAPN and Ang II infused with indomethacin administered (n = 11) mice. One-way ANOVA with Tukey’s multiple comparisons test. *P < 0.05 (**b**) Scatter dot plots showing the means ± SD of the fold change of transendothelial migration activity of splenic monocytes stimulated with 100 μM BAPN and 1 ng/ml Ang II in the presence or absence of 10^−6^ M indomethacin from that of unstimulated monocytes. *P < 0.05 n = 6 per group. Four outliers were identified and removed by the ROUT function on GraphPad Prism prior to Mann-Whitney U Test. (**c**) Scatter dot plots showing the means ± SD of PECAM-1 expression on peripheral blood monocytes measured as mean fluorescence intensity. Peripheral blood was collected after 4 hours of BAPN/Ang II administration and processed for flow cytometry. Vehicle group: n = 5. Indomethacin group: n = 5. Mann-Whitney U Test. *P < 0.05 (**d**) Scatter dot plots showing the means ± SD of PECAM-1 expression on aortic endothelial cells measured as mean fluorescent intensity. Cells stained with tomato lectin were measured for fluorescence intensity for PECAM-1 (n = 5). Mann-Whitney U Test.

As monocytes are known to migrate toward C-C motif chemokine ligands 2, 7, and 12 (CCL2, CCL7, and CCL12)^11,12^, we investigated whether indomethacin is capable of downregulating CCL2, CCL7, and CCL12 production in aortas. Real-time PCR analysis showed that administration of BAPN/Ang II significantly increased *Ccl2* and *Ccl7* mRNA expression in the aorta; however, *Ccl2* and *Ccl7* expression were not downregulated by indomethacin administration in mice infused with BAPN/Ang II (Fig. 5a).

We then investigated *in vitro* whether indomethacin blocks transendothelial migration of monocytes. Monocytes were isolated from the spleen by negative selection using antibodies against T-cell, B-cell, natural-killer-cell, DC, and erythrocyte markers as well as anti-IE/IA (major histocompatibility complex class II) antibodies for exclusion of macrophages^13–15^ and anti-CD45 and anti-F4/80 antibodies for subsequent positive selection. Then, we placed the isolated monocytes above a layer of TKD2 endothelial cells in the upper chamber of a trans-well plate which contains BAPN/Ang II and counted the number of the monocytes that migrated to the bottom chamber containing CCL2. We chose CCL2 since its involvement in aortic dissection has been observed in multiple reports^8,16,17^. With the addition of indomethacin in the upper chamber, the monocytes present in the lower chamber were half that migrating through the layer of endothelial cells in the absence of indomethacin (Fig. 5b). Since previous studies have suggested that downregulation of platelet endothelial cell adhesion molecule 1 (PECAM-1) by indomethacin is responsible for lower transendothelial migration activity^18,19^, we monitored the surface expression of PECAM-1 on peripheral blood monocytes using flow cytometry. We collected peripheral blood 4 hours after the initiation of BAPN subcutaneous injection and Ang II infusion to see whether PECAM-1 expression is reduced prior to the initiation of aortic dissection. Although both vehicle-treated and indomethacin-treated groups did not develop dissection in 4 hours, the indomethacin-treated mice showed decreased PECAM-1 expression compared to the vehicle-treated mice (Fig. 5c and Supplementary Fig. 3). Fluorescence intensity analysis of aortic sections showed endothelial cells (cells stained by tomato lectin^20^) from vehicle-administered and indomethacin-administered BAPN/Ang II-infused mice expressed comparable levels of PECAM-1 (Fig. 5d and Supplementary Fig. 4). These results imply that indomethacin administration blocks transendothelial migration of peripheral blood monocytes to the abdominal aortic wall by downregulating PECAM-1 expression.

## Discussion

There is an increasing appreciation of the involvement of inflammation in development and progression of aortic dissection^1,4,8,21,22^. We previously reported that granulocyte-macrophage colony-stimulating factor and macrophage infiltration into the aortic lesion site is required for aortic dissection formation in a mouse model of aortic dissection^1^. Neutrophil infiltration derived by C-X-C motif chemokine ligand 1 (CXCL1) has also been reported to cause progression from aortic dissection to aortic rupture in mice fed beta-aminopropionitrile (BAPN) and infused with angiotensin II (Ang II)^4^. Consistent with this, patients with aortic dissection show an increase in inflammatory biomarkers including peripheral blood natural killer cells, B cells, regulatory T cells, C-reactive protein, and cytokines such as interleukin-6 (IL-6) and -8 (IL-8), tumour necrosis factor α (TNFα), and C-C motif chemokine ligand 2 (CCL2)^22^.

The present study reports that an anti-inflammatory agent, indomethacin, protects against aortic dissection in a recently described murine model of the condition involving BAPN and Ang II administration^6^. We demonstrated that indomethacin administration was associated with reduced incidence of aortic dissection and less fatal abdominal aortic rupture, and a decrease in monocyte/macrophage accumulation to the abdominal aortic wall. We also showed that the reduction of the monocyte/macrophage population in the abdominal aortic wall correlated with impaired transendothelial migration and reduced surface expression of platelet endothelial cell adhesion molecule-1 (PECAM-1) on monocytes but not on abdominal vascular endothelial cells.

The murine model of aortic dissection using BAPN/Ang II highlights different pathologies in the thoracic and abdominal aorta as observed in the present study. Distinct immune cell distribution and morphology in the thoracic and abdominal aortas have been observed^5^. A different model of aortic dissection/aneurysm using fibrillin-1 knockout mice contributes to the development of thoracic aortic dissection/aneurysm with increase in transforming growth factor beta (TGF-β) pathway activity^23^. On the other hand, administration of TGF-β neutralizing antibody is known to induce abdominal aortic dissection^9^. Furthermore, thoracic vascular smooth muscle cells (VSMCs) show different embryological origin from abdominal VSMCs, and VSMCs show conflicting response to TGF-β depending on their embryological origin^24^. Thus, the difference in efficacy of indomethacin administration in thoracic and abdominal aortas could also arise from the VSMC lineage diversity as well as other still undefined differences.

Mechanistically, monocytes/macrophages in the aortic wall were significantly decreased in mice administered indomethacin with BAPN/Ang II infusion even though neutrophil and T cell accumulation in the aortic wall from the indomethacin-administered group was not significantly lower than that from the vehicle-administered group. This was supported by immunohistochemical analysis showing less accumulation of Mac-3-positive monocytes/macrophages in the abdominal aortic wall from the mice receiving BAPN/Ang II infusion with indomethacin administration. Moreover, improved survival and decreased incidence of aortic dissection were associated with depletion of monocytes/macrophages. Since neutrophil accumulation was comparably high in the vehicle-administered and indomethacin-administered groups, at least the earlier events of aortic dissection appeared similar. These results together suggest that indomethacin moderates evolution of intramural processes through attenuation of monocyte/macrophage accumulation in the aortic wall as early events seemed to be nearly identical.

In aortic aneurysms and atherosclerosis, previous reports have indicated that peripheral blood monocytes are recruited to the aorta^10,25,26^. However, to the best of our knowledge, this study is the first to show that reduction in transendothelial migration activity of peripheral blood monocyte correlates with decrease in the incidence of aortic dissection. The indomethacin-administered BAPN/Ang II-infused mice showed twice as many peripheral blood monocytes in percentages as the vehicle-administered BAPN/Ang II-infused mice. Our data and previous reports suggest that a possible mechanism is attenuated migration of monocytes from peripheral blood to the aortic wall. CCL2, CCL7, and CCL12 are major chemoattractants for monocytes^11,12^ and CCL2 deficiency has been reported to result in attenuated accumulation of macrophages in an atherosclerosis model^27^. We observed increases in *Ccl2* and *Ccl7* mRNA expression in the aorta of mice infused with BAPN/Ang II. However, administration of indomethacin did not decrease *Ccl2* and *Ccl7* expression in the aorta of mice infused with BAPN/Ang II. The expression of *Ccl12* in vehicle- and indomethacin-administered BAPN/Ang II infused mice was comparable to that of sham mice. Thus, CCL2, CCL7, and CCL12 were not likely to be directly involved in mitigation of abdominal aortic dissection by indomethacin.

Furthermore, monocytes stimulated by BAPN/Ang II exhibited transendothelial migration activity toward CCL2 *in vitro*, whereas indomethacin reduced transendothelial migration activity of monocytes stimulated by BAPN/Ang II by 50%. Neutralization of surface molecules such as PECAM-1^18^, CD99^28^, CD157^29^, lymphocyte function-associated antigen 1 (LFA-1), macrophage-1 antigen (Mac-1), or very late antigen-4 (VLA-4)^30^ has been shown to attenuate transendothelial migration of monocytes. Of these molecules, PECAM-1 is involved in the last stage of transendothelial migration, and only PECAM-1 expression is known to be downregulated by indomethacin or a cyclooxygenase-2 specific inhibitor via blocking prostaglandin E_2_ (PGE_2_) production^19^. It has also been reported that PGE_2_ regulates PECAM-1 expression via nuclear factor kappa-light-chain-enhancer of activated B cells (NF-kB)^19^, and PGE_2_ has been shown to regulate the transcriptional activity of NF-kB^31^ which has two consensus sites in PECAM-1 promoter^32^ and regulate PECAM-1 transcriptional activity^33^. In the present study, we demonstrated PECAM-1 expression by peripheral blood monocytes was reduced by indomethacin administration in mice receiving BAPN/Ang II infusion before the development of aortic dissection. On the other hand, PECAM-1 expression on endothelial cells was not significantly affected by indomethacin administration. Therefore, this suggests that indomethacin impairs transendothelial migration through downregulation of PECAM-1. In order to further address whether attenuating transendothelial migration by PECAM-1 downregulation in monocytes leads to partial blockade of monocyte/macrophage accumulation in the abdominal aortic wall, a study *in vivo* using monocyte specific PECAM-1 knockout mice is needed. Furthermore, transendothelial migration involves multiple steps, and it needs to be investigated whether indomethacin affects other molecules involved in transendothelial migration such as CD99^28^, CD157^29^, LFA-1, Mac-1, or VLA-4^30^ in order to understand the whole process of transendothelial migration attenuation by indomethacin. In addition, indomethacin is known to inhibit PGE_2_^34^ and prostaglandin F_2α_ (PGF_2α_) synthesis^35^, and antagonizing the PGE_2_ receptor subtype 4 (EP4) and PGF_2α_ receptor (FP) have been shown to attenuate aortic dissection^36^ and aneurysm in mice^37^, respectively. Macrophage infiltration was observed in dissected aortas induced by antagonizing EP4 similarly to our observation^36^. It is possible that inhibition of activation of multiple prostaglandin receptors by indomethacin precedes downregulation of PECAM-1.

We did not observe increase in the DC population in the aorta nor peripheral blood, and therefore did not further pursue effects on DC subsets (i.e. plasmacytoid, conventional, and monocyte-derived). Self-renewing resident arterial macrophages have also recently been identified^38^. It is possible that indomethacin affects the function, number, and proliferation capacity of the arterial resident macrophages as well.

In summary, we demonstrated that indomethacin administration was associated with reduced rates of aortic dissection as evidenced by decreased incidence and mortality in a murine model of the condition. Indomethacin administration decreased monocyte/macrophage accumulation in the abdominal aortic wall and reduced monocytic transendothelial migration activity.

## Methods

### Mice

All mice used in this study were C57BL/6J mice 5 to 6 weeks of age purchased from CLEA or SLC Japan. Experiments in this study comply with the Jichi Medical University Guide for Laboratory Animals and the Guide for the Care and Use of Laboratory Animals published by the U.S. National Institutes of Health (NIH Publication, eighth edition, 2011). All animal protocols were approved by the Institutional Review Committee of Jichi Medical University.

### Aortic dissection model

Aortic dissection was induced in mice based on a previously published method using administration of beta-aminopropionitrile (BAPN)^5,6^. Osmotic pump models 2002 and 1002 (Alzet) were filled with BAPN (Sigma-Aldrich A3134) and angiotensin II (Ang II; Peptide Institute 4001), respectively, and incubated in saline at 37 °C for 4 hours to overnight before implantation. The concentrations of BAPN and Ang II were adjusted so that BAPN and Ang II were infused at the rates of 150 mg/kg/day and 1000 ng/kg/min. Before implantation of osmotic pumps, 6 mg/kg of pentobarbital was intraperitoneally injected. Absence of pedal reflex was used as the indicator of deep anaesthesia. After implantation, mice were allowed to rest on a heating pad until recovering and becoming alert. Osmotic pump models 2002 and 1002 filled with distilled water and 10 mM acetic acid in saline were implanted to sham mice. Mice were observed for 2 weeks to monitor survival and incidence rates. Mice surviving for 2 weeks were euthanised by intraperitoneal injection of 100 mg/kg pentobarbital, and aortas were removed. The photographs of aortas were captured at 6.3× magnification with a camera (Leica, MC170 HD) mounted on a stereo-microscope (Leica, MZ75). For analysis before dissection, BAPN was injected subcutaneously and the osmotic pump model 1003D filled with Ang II was implanted. After 4 hours, the mice were euthanised.

### Indomethacin administration

Indomethacin (Sigma-Aldrich I7378) was dissolved in drinking water at a concentration of 12 mg/L. The solution was provided ad libitum to mice 3 days before the initiation of BAPN/Ang II infusion and exchanged to fresh solution every 2 to 3 days. All mice were kept in isolation during the indomethacin or vehicle administration period to ensure comparable volumes of water intake. Each mice ingested an average of 83.97 µg/day of indomethacin.

### Cell isolation for flow cytometry

After 7 days of BAPN/Ang II administration (or 10 mM acetic acid in saline and water administration for the sham group), mice were euthanised, and the aortic wall was removed and digested after blood was drawn. The aortic wall was digested according to a previously published method with some modification^39^. After the removal of periaortic fat tissue and lymph nodes, the aorta inferior to the diaphragm and superior to the bifurcation was cut into <5 mm pieces prior to enzymatic digestion on 6 well-plates. Two ml of digestion enzyme solution (125 U/ml collagenase type XI, 60 U/ml hyaluronidase type I-s, 60 U/ml DNase I, and 450 U/ml collagenase type I in PBS) was added to each well. The aortas were digested for 1 hour at 37 °C with gentle agitation. Single cells were collected in 50-mL test tubes by running digested aortas through 70 μm cell strainers, and the digested aorta remaining on the filter was sheared and collected in the same tubes by running FACS buffer through the filters. Peripheral blood was drawn with a heparinised syringe and collected in a heparinised 1.5 mL tube. Red blood cells were lysed in BD Pharm Lyse™ (BD, 555899) for 15 minutes at room temperature, and then washed twice with PBS containing 5% FCS and 2 mM EDTA. Prior to antibody labelling, the number of leukocytes from 500 µl of peripheral blood were counted by Countess (Invitrogen).

### Antibody labelling for flow cytometry

Single cells from the aortic wall or peripheral blood were pelleted by centrifugation at 1300 rpm for 5 minutes at 4 °C. Each pellet was resuspended in 1 mL of FACS buffer and transferred to a 1.5-mL tube. Cell suspensions were pelleted again by centrifugation. In order to prevent antibodies from non-specifically binding to Fc receptors, the pellets were resuspended in FACS buffer containing anti-mouse CD16/32 antibody (BioLegend, TrueStain fcX) and incubated for 15 minutes at room temperature. Peripheral whole blood was mixed in BD Pharm Lyse™ containing anti-mouse CD16/32 antibody and incubated for 15 minutes at room temperature and then pelleted by centrifugation at 1300 rpm for 5 minutes at 4 °C. Cell suspensions were mixed with an antibody cocktail containing anti-mouse CD3e antibody conjugated with FITC (BioLegend, 145-2C11), anti-mouse CD45 antibody conjugated with Alexa Flour 700 (BioLegend, 30-F11), anti-mouse CD11b antibody conjugated with pacific blue (BioLegend, M1/70), anti-mouse CD11c antibody conjugated with APC (BioLegend, N418), and anti-mouse Ly6G antibody conjugated with APC-Cy7 (BioLegend, 1A8) for the post-dissection analysis or anti-mouse Ly6G antibody conjugated with FITC (BioLegend, 1A8), anti-mouse CD45 antibody conjugated with Alexa Flour 700 (BioLegend, 30-F11), anti-mouse CD11b antibody conjugated with pacific blue (BioLegend, M1/70), anti-mouse PECAM-1 antibody conjugated with APC (BioLegend, MEC13.3), and anti-mouse CD11c antibody conjugated with APC-Cy7 (BioLegend, N418) for the pre-dissection analysis and incubated for 30 minutes at 4 °C in the dark. Cells were washed twice and re-suspended in FACS buffer containing propidium iodide. All data were obtained on a Sony SH800 flow cytometer and analysed with FlowJo (FlowJo, LLC).

### Immunohistochemistry

Aortas were fixed in Ufix (Sakura Finetek) overnight and then washed with PBS. The fixed aortas were embedded in paraffin, and then the paraffin blocks were sliced into 5 μm sections. The sections were stretched in a 40 °C water bath and dried on glass slides overnight. After deparaffinization and rehydration, the sections were heated in citric acid in a microwave for 7 minutes. Endogenous peroxidase activity was quenched with 3% H_2_O_2_ for 20 minutes. After 20 minutes of incubation with 2.5% normal goat serum for blocking non-specific binding, the sections were incubated overnight with rat anti-mouse Mac-3 antibody (BD M3/84) diluted to 1:200 in SignalStain® (CST). Sections were washed three times with TBST before and after incubation with anti-rat IgG conjugated with horseradish peroxidase micropolymers (ImmPRESS™ REAGENT Kit anti-rat IgG, Vector) for 30 minutes. ImmPACT™ DAB peroxidase substrate (Vector) was applied for 2 minutes before rinsing with distilled water. Sections were counterstained with haematoxylin for 15 minutes and differentiated in 1% hydrochloric acid in 70% ethanol and then mounted in VectaMount™ (Vector). BZX-700 (KEYENCE) was used to capture images. Photographs of the aortas from sham mice were taken at 200× magnification, and those from BAPN/Ang II-infused mice with or without indomethacin administration were taken at 200× magnification and stitched with BZ-II Analyzer (KEYENCE, BZ-H2A). For fluorescent immunohistochemistry, after antigen retrieval, the sections blocked with 2.5% normal goat serum for 20 min were incubated with anti-PECAM-1 antibody diluted to 1:10 in antigen-antibody reaction enhancing buffer (Setsuyaku-kun, DRC, Japan). After 2 hours of incubation, the sections were washed with PBS three times and then incubated for 1 hour with anti-rabbit IgG antibodies conjugated with Alxa 488 (Thermo Fisher Scientific) diluted to 1:100 and 20 μg/ml DyLight 594 labeled *Lycopersicon Esculentum* Lectin (Tomato lectin) (Vector) in the antigen-antibody reaction enhancing buffer. The sections were washed in PBS twice after incubated for an hour, and stained with 0.5 μg/ml 4′,6-diamidino-2-phenylindole for 10 minutes. After washing in PBS three times, the sections were mounted in Fluorescence Mounting Medium (Dako). Fluorescent images were captured by microscopy (BX63, Olympus) at 400× magnification. Mean fluorescence intensity in the green fluorescence channel on regions stained with DyLight 594 labelled lectin was obtained by Image-J.

### Elastica van gieson staining

After deparaffinisation and rehydration, sections were stained in Maeda’s Resorcin-Fuchsin (Muto Pure Chemicals) for 1 hour. The sections were differentiated in 100% ethanol three times and washed in running tap water for 5 minutes then stained in Weigert’s iron haematoxylin for 5 minutes. After washing in running tap water for 20 minutes, the sections were stained in Van Gieson’s stain with 0.03% Sirius red for 10 minutes. Excess stain solution was removed by rapidly rinsing the sections with water. After dehydration and clearing, the sections were mounted with VectaMount® permanent mounting medium (Vector). BZX-700 (KEYENCE) was used to capture images. Photographs of the aortas from sham mice were taken at 200× magnification, and those from BAPN/Ang II-infused mice with or without indomethacin administration were taken at 200× magnification and stitched with BZ-II Analyzer (KEYENCE, BZ-H2A).

### Clodronate administration

Mice were injected intraperitoneally with 8 ml/kg of empty anionic liposomes or anionic liposomes encapsulating clodronate (FormuMax Scientific Inc.) 2 days before and 7 days after the initiation of BAPN/Ang II infusion.

### Real-time PCR

The aortic wall inferior to the diaphragm and superior to the bifurcation was stored in RNA later at 4 °C overnight. The aortic wall was homogenised in the Bioprep-24 Homogenizer system (Bio Medical Science, Japan) on the following day. Total RNA was purified with RNeasy® (Qiagen) according to the manufacturer’s instructions. ReverTra Ace® (TOYOBO) was used for reverse transcription of total RNA. Resulting cDNA was mixed with THUNDERBIRD® SYBR® qPCR Mix (TOYOBO) and a primer set. Real-time PCR reaction was run in a LightCycler® 480 Instrument II (Roche). The reaction mix was pre-incubated at 95 °C for 30 seconds with amplification cycle of 95 °C for 5 seconds, 56 °C or 60 °C for 10 seconds, and 72 °C for 30 seconds which was repeated 40 times. Following the amplification cycle, the reaction mix was run for melt curve analysis. As described previously^40^, the reference genes, *Cdc 40*, *Nup88*, *Sppl2*, and *Wbp4* were identified by the RefGenes database of Genevestigator software (Nebion) in combination with BestKeeper© software^41^. The Ct value of *Ccl2* was normalised to the geometric mean of the Ct values of these reference genes. ΔCt was calculated as subtraction of Ct_(*Ccl2*)_ from Ct_(*reference gene*)_. ΔCt of each group was compared and tested for statistical significance^42^. The primers were as follows: *Ccl2* forward AGCTGTAGTTTTTGTCACCAAGC; reverse GTGCTGAAGACCTTAGGGCA; *Ccl7* forward GTCCCTGGGAAGCTGTTATCTTCAA; reverse GACCCACTTCTGATGGGCTT; *Ccl12* forward GGAAGCTGTGATCTTCAGGAC; reverse GGGGAACTTCAGGGGGAAATA; *Cdc40* forward TGATCGGCATCTGGGGGCTG; *Cdc40* reverse GGAGACAAAGTCACTGCGGGC; *Sppl2* forward TGACCTCAGCAAAGTGTCTCTCCT; *Sppl2* reverse GATTTGGCTCCCCCTCCCGA; Nup88 forward GATTTGGCTCCCCCTCCCGA; *Nup88* reverse TTTGACCCTCCGCTGAATCTCC; *Wbp4* forward GGAGGGGAAGAGGCGGTGACA; *Wbp4* reverse ACTTCCAGTAGTCGGCCATGACG

### Transendothelial migration

ThinCert™ cell culture inserts (Greiner Bio-One) were coated with gelatine and 7.5 × 10^4^ of the TKD2 endothelial cell line^43^ was seeded prior to placing monocytes. After the endothelial cells formed a tight layer, monocytes were isolated from mouse spleens based on a previously published method^44,45^. Splenic cells were squeezed out of the spleen with curved forceps and passed through a 26G needle several times. Then, the erythrocytes were lysed in BD Pharm Lyse™ for 15 minutes. Fc receptors were simultaneously blocked by anti-mouse CD16/32 antibodies (BioLegend, TrueStain fcX) in BD Pharm Lyse™. Cells were pelleted and washed once with MACS® buffer (Miltenyi Biotec). After the resuspension, cells were incubated with biotin conjugated antibodies against the following murine antigens: CD3ε (BioLegend, 145-2C11), CD45R/B220 (BioLegend, RA3-6B2), TER-119 (BioLegend, TER-119), IA/IE (BioLegend, M5/114.15.2), CD49b (BioLegend, DX5), Ly6G (BioLegend, 1A8), and CD193 (BioLegend, J073E5) at 4 °C for 30 minutes. After washing, the labelled cells were incubated with anti-biotin microbeads (Miltenyi Biotec) at 4 °C for 15 minutes. The labelled cells were washed once prior to magnetic separation in Auto MACS® (Miltenyi Biotec). The depletion program on autoMACS® was used and the depletion of labelled cells was run twice. The negatively selected cells were washed once and labelled with streptavidin conjugated with APC, AF700 anti-CD45 antibody (BioLegend, 30-F11), and PE-Cy7 anti-F4/80 antibody (eBioscience, BM8) after 15 minutes of incubation at 4 °C. Prior to flow cytometry analysis and sorting, the labelled cells were washed once and propidium iodide was added to aid in removing dead cells. Propidium iodide- and APC-negative, and CD45- and F4/80-positive cells were sorted by the SH800 cell sorter (Sony). 3 × 10^4^ to 2 × 10^5^ cells were placed in the inserts that had formed a layer of endothelial cells with 100 μM BAPN and 1 ng/ml Ang II with or without 10 M^−6^ indomethacin. The inserts were placed on RPMI containing 100 μg/ml CCL2 (Peprotech) in a 24-well plate and incubated for 120 minutes. The number of monocytes migrating to the well were counted, and percentages of monocytes that migrated was calculated by dividing the number of migrated monocytes by the total number of monocytes. To minimise intra-assay variation, the results of each group were normalised with the percentage of monocytes that migrated without BAPN and Ang II. TKD2 cells were obtained from Japanese Collection of Research Bioresources Cell Bank (Osaka, Japan).

### Statistical analysis

The normality of each data set was tested by the Kolmogorov–Smirnov test prior to assessment of statistical significance. The statistical test used in each experiment is indicated in the figure legends. *P* values less than 0.05 were considered significant.

## Supplementary information

Supplementary Material

## Data Availability

The datasets generated during and/or analysed during the current study are available from the corresponding author on reasonable request.

## References

[1] Son BK (2015). Granulocyte macrophage colony-stimulating factor is required for aortic dissection/intramural haematoma. Nat. Commun..

[2] Hagan PG (2000). The International Registry of Acute Aortic Dissection (IRAD): new insights into an old disease. JAMA.

[3] Tran TP, Khoynezhad A (2009). Current management of type B aortic dissection. Vasc. Health Risk Manag..

[4] Anzai A (2015). Adventitial CXCL1/G-CSF expression in response to acute aortic dissection triggers local neutrophil recruitment and activation leading to aortic rupture. Circ. Res..

[5] Kanematsu Y (2010). Pharmacologically induced thoracic and abdominal aortic aneurysms in mice. Hypertension.

[6] Logghe G (2018). Propagation-based phase-contrast synchrotron imaging of aortic dissection in mice: from individual elastic lamella to 3D analysis. Sci. Rep..

[7] Li JS, Li HY, Wang L, Zhang L, Jing ZP (2013). Comparison of β-aminopropionitrile-induced aortic dissection model in rats by different administration and dosage. Vascular.

[8] Ju X (2013). Interleukin-6-signal transducer and activator of transcription-3 signaling mediates aortic dissections induced by angiotensin II via the T-helper lymphocyte 17-interleukin 17 axis in C57BL/6 mice. Arterioscler. Thromb. Vasc. Biol..

[9] Wang Y (2010). TGF-beta activity protects against inflammatory aortic aneurysm progression and complications in angiotensin II-infused mice. J. Clin. Invest..

[10] Mellak S (2015). Angiotensin II mobilizes spleen monocytes to promote the development of abdominal aortic aneurysm in Apoe−/− mice. Arterioscler. Thromb. Vasc. Biol..

[11] Deshmane SL, Kremlev S, Amini S, Sawaya BE (2009). Monocyte chemoattractant protein-1 (MCP-1): an overview. J. Interferon Cytokine Res..

[12] Lim JK (2011). Chemokine receptor Ccr2 is critical for monocyte accumulation and survival in West Nile virus encephalitis. J. Immunol..

[13] Franken L (2015). Splenic red pulp macrophages are intrinsically superparamagnetic and contaminate magnetic cell isolates. Sci. Rep..

[14] Kurotaki D (2011). CSF-1-dependent red pulp macrophages regulate CD4 T cell responses. J. Immunol..

[15] Kurotaki D, Uede T, Tamura T (2015). Functions and development of red pulp macrophages. Microbiol. Immunol..

[16] Tieu BC (2009). An adventitial IL-6/MCP1 amplification loop accelerates macrophage-mediated vascular inflammation leading to aortic dissection in mice. J. Clin. Invest..

[17] Martorell S (2016). Vitamin D Receptor Activation Reduces Angiotensin-II-Induced Dissecting Abdominal Aortic Aneurysm in Apolipoprotein E-Knockout Mice. Arterioscler. Thromb. Vasc. Biol..

[18] Muller WA, Weigl SA, Deng X, Phillips DM (1993). PECAM-1 is required for transendothelial migration of leukocytes. J. Exp. Med..

[19] Gong N, Wei H, Chowdhury SH, Chatterjee S (2004). Lactosylceramide recruits PKCalpha/epsilon and phospholipase A2 to stimulate PECAM-1 expression in human monocytes and adhesion to endothelial cells. Proc. Natl. Acad. Sci. USA.

[20] Villacampa, N., Almolda, B., González, B. & Castellano, B. Tomato lectin histochemistry for microglial visualization. *Methods Mol. Bio**l*. **1041** (2013). 10.1007/978-1-62703-520-0_23 23813385

[21] Luo F, Zhou XL, Li JJ, Hui RT (2009). Inflammatory response is associated with aortic dissection. Ageing Res. Rev..

[22] del Porto F (2010). Inflammation and immune response in acute aortic dissection. Ann. Med..

[23] Dietz HC, Loeys B, Carta L, Ramirez F (2005). Recent progress towards a molecular understanding of Marfan syndrome. Am. J. Med. Genet. C Semin. Med. Genet..

[24] Topouzis S, Majesky MW (1996). Smooth muscle lineage diversity in the chick embryo. Two types of aortic smooth muscle cell differ in growth and receptor-mediated transcriptional responses to transforming growth factor-beta. Dev. Biol..

[25] Swirski FK (2006). Monocyte accumulation in mouse atherogenesis is progressive and proportional to extent of disease. Proc. Natl. Acad. Sci. USA.

[26] Samadzadeh KM (2014). Monocyte activity is linked with abdominal aortic aneurysm diameter. J. Surg. Res..

[27] Gu L (1998). Absence of monocyte chemoattractant protein-1 reduces atherosclerosis in low density lipoprotein receptor-deficient mice. Mol. Cell.

[28] Schenkel AR, Mamdouh Z, Chen X, Liebman RM, Muller WA (2002). CD99 plays a major role in the migration of monocytes through endothelial junctions. Nat. Immunol..

[29] Lo Buono N (2011). The CD157-integrin partnership controls transendothelial migration and adhesion of human monocytes. J. Biol. Chem..

[30] Meerschaert J, Furie MB (1995). The adhesion molecules used by monocytes for migration across endothelium include CD11a/CD18, CD11b/CD18, and VLA-4 on monocytes and ICAM-1, VCAM-1, and other ligands on endothelium. J. Immunol..

[31] Poligone B, Baldwin AS (2001). Positive and negative regulation of NF-kappaB by COX-2: roles of different prostaglandins. J. Biol. Chem..

[32] Almendro N (1996). Cloning of the human platelet endothelial cell adhesion molecule-1 promoter and its tissue-specific expression. Structural and functional characterization. J. Immunol..

[33] Botella LM (2000). Identification of a functional NF-kappa B site in the platelet endothelial cell adhesion molecule-1 promoter. J. Immunol..

[34] Tanaka A, Hase S, Miyazawa T, Takeuchi K (2002). Up-regulation of cyclooxygenase-2 by inhibition of cyclooxygenase-1: a key to nonsteroidal anti-inflammatory drug-induced intestinal damage. J. Pharmacol. Exp. Ther..

[35] Farida Khanum A, Hai MA, Choudhury SA (1981). Effects of prostaglandin F2 alpha and its synthesis inhibitor indomethacin on corporaluteal functions in pseudopregnant rats. Bangladesh. Med. Res. Counc. Bull..

[36] Cao RY (2012). Prostaglandin receptor EP4 in abdominal aortic aneurysms. Am. J. Pathol..

[37] Fukuda M (2014). Exacerbation of intracranial aneurysm and aortic dissection in hypertensive rat treated with the prostaglandin F-receptor antagonist AS604872. J. Pharmacol. Sci..

[38] Ensan S (2016). Self-renewing resident arterial macrophages arise from embryonic CX3CR1(+) precursors and circulating monocytes immediately after birth. Nat. Immunol..

[39] Butcher, M. J., Herre, M., Ley, K. & Galkina, E. Flow cytometry analysis of immune cells within murine aortas. *J. Vis. Exp*., 10.3791/2848 (2011). 10.3791/2848 PMC3196167 21750492

[40] Hruz T (2011). RefGenes: identification of reliable and condition specific reference genes for RT-qPCR data normalization. BMC Genomics.

[41] Pfaffl MW, Tichopad A, Prgomet C, Neuvians TP (2004). Determination of stable housekeeping genes, differentially regulated target genes and sample integrity: BestKeeper–Excel-based tool using pair-wise correlations. Biotechnol Lett.

[42] Yuan JS, Reed A, Chen F, Stewart CN (2006). Statistical analysis of real-time PCR data. BMC Bioinformatics.

[43] Yanai N (1991). A tubule cell line established from transgenic mice harboring temperature-sensitive simian virus 40 large T-antigen gene. Jpn. J. Cancer Res..

[44] Swirski FK (2009). Identification of splenic reservoir monocytes and their deployment to inflammatory sites. Science.

[45] Houthuys E, Movahedi K, De Baetselier P, Van Ginderachter JA, Brouckaert P (2010). A method for the isolation and purification of mouse peripheral blood monocytes. J. Immunol. Methods..

