# Supplementary material for: Indomethacin reduces rates of aortic dissection and rupture of the abdominal aorta by inhibiting monocyte/macrophage accumulation in a murine model
Source: Sci Rep. 2019 Jul 24;9:10751. doi: 10.1038/s41598-019-46673-z (PMC6656736; doi:10.1038/s41598-019-46673-z)
Supplement: Supplementary file 1 — Supplementary Material [file 41598_2019_46673_MOESM1_ESM.pdf]

Supplementary Materials for

Indomethacin reduces rates of aortic dissection of the abdominal aorta and rupture by inhibiting monocyte/macrophage accumulation in a murine model

Shota Tomida[1], Kenichi Aizawa[1], Norifumi Nishida[2], Hiroki Aoki[3], Yasushi Imai[1], Ryozo Nagai[6], Toru Suzuki[4,5,6]*

1 Department of Clinical Pharmacology, Jichi Medical University, Tochigi, Japan

2 Division of Cardiovascular Medicine, Department of Internal Medicine, Kurume University School of Medicine, Kurume, Fukuoka, Japan

3 Cardiovascular Research Institute, Kurume University, Kurume, Japan

4 Department of Cardiovascular Sciences, University of Leicester, Cardiovascular Research Centre, Leicester, UK

5 National Institute for Health Research Leicester Cardiovascular Biomedical Research Unit, Glenfield Hospital, Leicester, UK

6 Jichi Medical University, Tochigi, Japan

Supplementary Figure 1

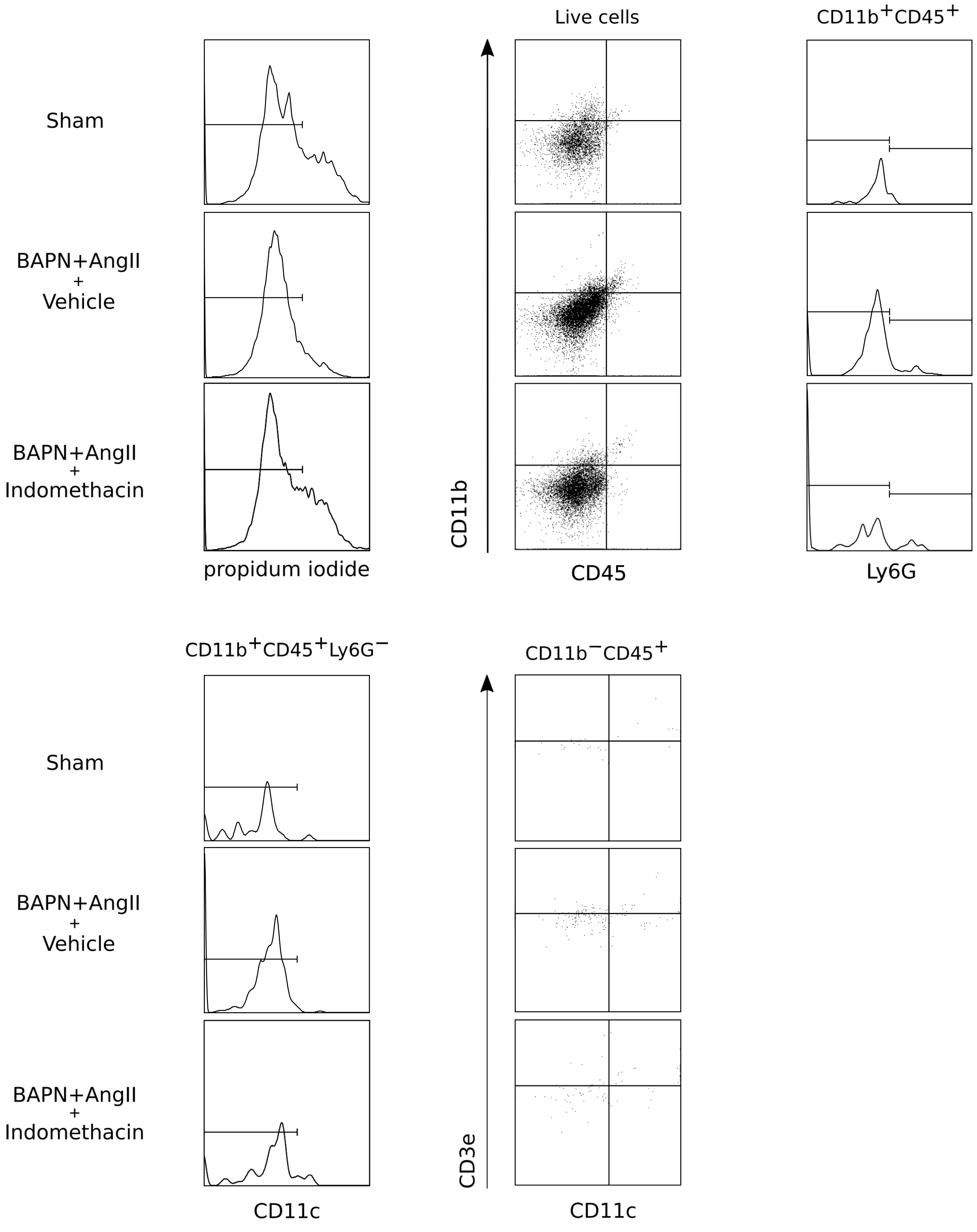

Supplementary Figure 1. Representative dot plots of flow cytometry analysis on cells from the abdominal aortic wall

After 7 days of BAPN and Ang II infusion with or without indomethacin administration, cells from the aortic wall were analysed by flow cytometry to identify and measure ratios of neutrophils ($CD45^{+}$ $CD11b^{+}$ $Ly6G^{+}$), monocytes/macrophages ($CD45^{+}$ $CD11b^{+}$ $CD11c^{-}$ $Ly6G^{-}$), dendritic cells (DCs) ($CD45^{+}$ $CD11b^{-}$ $CD11c^{+}$), and T cells ($CD45^{+}$ $CD11b^{-}$ $CD3^{+}$) to the total live cells. After exclusion of cells stained by propidium iodide, $CD45^{+}$ $CD11b^{+}$ and $CD45^{+}$ $CD11b^{-}$ populations were gated. In $CD45^{+}$ $CD11b^{+}$ populations, $Ly6G^{+}$ populations were identified as neutrophils, and $Ly6G^{-}$ populations were further gated and were monitored for CD11c expression. Monocytes/macrophages were then identified as $CD11c^{-}$. In $CD45^{+}$ $CD11b^{-}$ populations, $CD3e^{+}$ cells were identified as T cells and $CD11c^{+}$ cells were identified as DCs, respectively.

Supplementary Figure 2

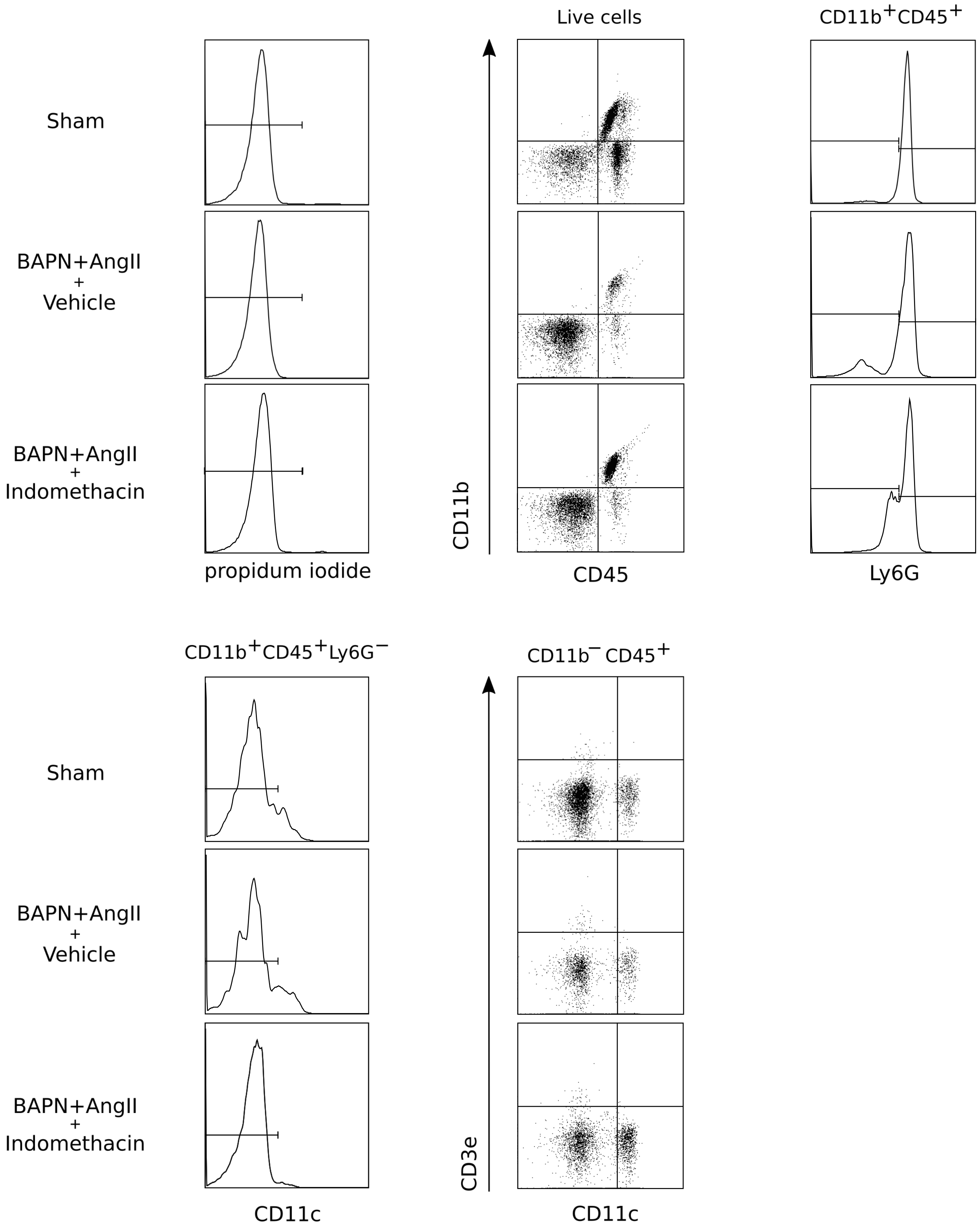

Supplementary Figure 2. Representative dot plots of flow cytometry analysis on peripheral blood leukocytes

After 7 days of BAPN and Ang II infusion with or without indomethacin administration, leukocytes from peripheral blood were analysed by flow cytometry to identify and measure ratios of neutrophils ($CD45^{+}$ $CD11b^{+}$ $Ly6G^{+}$), monocytes ($CD45^{+}$ $CD11b^{+}$ $CD11c^{-}$ $Ly6G^{-}$), dendritic cells (DCs) ($CD45^{+}$ $CD11b^{-}$ $CD11c^{+}$), and T cells ($CD45^{+}$ $CD11b^{-}$ $CD3^{+}$) to $CD45^{+}$ cells. After exclusion of cells stained by propidium iodide, $CD45^{+}$ $CD11b^{+}$ and $CD45^{+}$ $CD11b^{-}$ populations were gated. In $CD45^{+}$ $CD11b^{+}$ populations, $Ly6G^{+}$ populations were identified as neutrophils, and $Ly6G^{-}$ populations were further gated and were monitored for CD11c expression. Monocytes were then identified as $CD11c^{-}$. In $CD45^{+}$ $CD11b^{-}$ populations, $CD3e^{+}$ cells were identified as T cells and $CD11c^{+}$ cells were identified as DCs, respectively.

Supplementary Figure 3

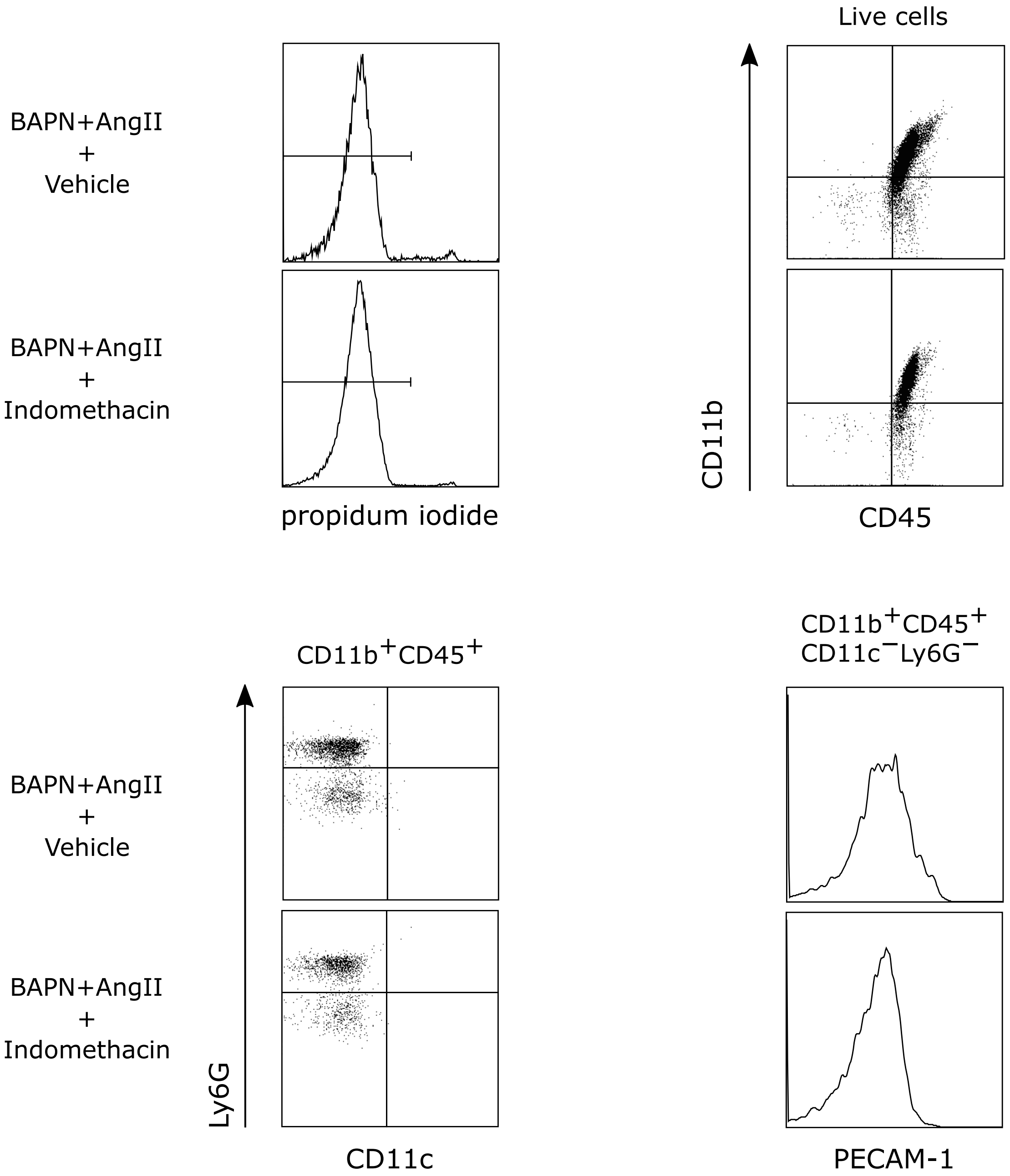

Supplementary Figure 3. Representative dot plots of flow cytometry analysis of platelet endothelial cell adhesion molecule-1 (PECAM-1) expression on peripheral blood monocytes

After 4 hours of BAPN/Ang II infusion with or without indomethacin administration, peripheral blood leukocytes were isolated and analysed. After exclusion of cells stained by propidium iodide, $CD45^{+}$ $CD11b^{+}$ were gated. In $CD45^{+}$ $CD11b^{+}$ populations, $Ly6G^{-}$ $CD11c^{-}$ populations were further gated, and PECAM-1 expression was measured.

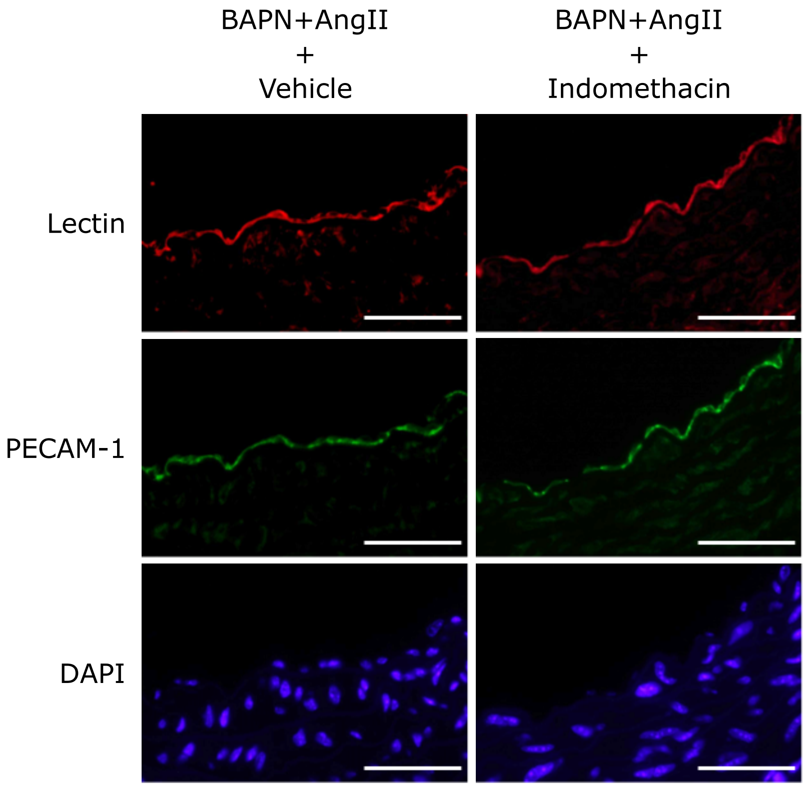
BAPN+AngII
+
Vehicle
BAPN+AngII
+
Indomethacin
Lectin
PECAM-1
DAPI

Supplementary Figure 4. Representative images of fluorescent immunohistochemistry

Sections from abdominal aortas were stained with anti-PECAM-1 antibody (green), tomato lectin (red), and 4',6-diamidino-2-phenylindole (DAPI) (blue). Fluorescence intensity of the green channel was measured on cells stained with tomato lectin. Scale bar: 50 µm.
